# Incident reporting systems: a comparative study of two hospital divisions

**DOI:** 10.1186/s13690-016-0146-8

**Published:** 2016-08-15

**Authors:** Tanya Hewitt, Samia Chreim, Alan Forster

**Affiliations:** 1 Population Health, University of Ottawa, 25 University Private, Ottawa, ON Canada K1N 7K4; 2 Telfer School of Management, University of Ottawa, 55 Laurier Avenue East, Ottawa, ON K1N 6N5 Canada; 3 Department of Medicine, Faculty of Medicine, University of Ottawa, Civic Campus, 1053 Carling Avenue, Box 684, Administrative Services Building, Ottawa, ON K1Y 4E9 Canada; 4 Ottawa Hospital Research Institute Ottawa Hospital, Civic Campus, 1053 Carling Avenue, Box 684, Administrative Services Building, Ottawa, ON K1Y 4E9 Canada

**Keywords:** Patient safety, Medical errors, Qualitative research, Internal medicine, Obstetrics, Neonatology

## Abstract

**Background:**

Previous studies of incident reporting in health care organizations have largely focused on single cases, and have usually attended to earlier stages of reporting. This is a comparative case study of two hospital divisions’ use of an incident reporting system, and considers the different stages in the process and the factors that help shape the process.

**Method:**

The data was comprised of 85 semi-structured interviews of health care practitioners in general internal medicine, obstetrics and neonatology; thematic analysis of the transcribed interviews was undertaken. Inductive and deductive themes are reported. This work is part of a larger qualitative study found elsewhere in the literature.

**Results:**

The findings showed that there were major differences between the two divisions in terms of: a) what comprised a typical report (outcome based vs communication and near-miss based); b) how the reports were investigated (individual manager vs interdisciplinary team); c) learning from reporting (interventions having ambiguous linkages to the reporting system vs interventions having clear linkages to reported incidents); and d) feedback (limited feedback vs multiple feedback).

**Conclusions:**

The differences between the two divisions can be explained in terms of: a) the influence of litigation on practice, b) the availability or lack of interprofessional training, and c) the introduction of the reporting system (top-down vs bottom-up approach). A model based on the findings portraying the influences on incident reporting and learning is provided. Implications for practice are addressed.

## Background

A number of studies have found high incidences of adverse events in health care. These include *To Err is Human* [1], the United Kingdom’s (UK’s) *An Organization with a Memory* [2] and the *Canadian Adverse Events Study* (CAES) [3]. Incident reporting has been recommended as one of several tools to address this patient safety problem [4]. Incident reporting systems (IRS) have met with some success. For example, Swartz [5] describes the success a hospital had with an electronic IRS which allowed key players greater access to information they needed to effect and prioritize corrective actions. Osmond et al. [6] noted the diversity of front line practitioner reported events in a successful Intensive Care Unit (ICU) IRS. Using a new human factors focus within an IRS, Morag et al. [7] reported very promising results. Overall, there have been several reports of success with various IRSs.

However, IRSs have been sharply criticized as well. Blais, Bruno, Bartlett, & Tamblyn [8] compared the chart review process against an incident reporting technique in adult medicine and surgery in hospitals in a province, and found that only 15 % of incidents in the chart review were identified in the IRS. Shojania [9] spoke of the “frustrating case of incident reporting systems”. He highlighted physician underreporting, the lack of a denominator in IRS metrics (incident reports reveal only how many incidents occurred, but do not capture how many could have occurred), and the deceiving metric of compliance with having an IRS irrespective of how the system functions (the system could be solely a data collection system without any follow up). In a later paper, Shojania further stated that relying on IRSs exclusively is not a good way to assess patient safety, but instead a number of different methods should be used [10]. In his report “Hospital Incident Reporting Systems Do Not Capture Most Patient Harm”, the Inspector General of the American Department of Health and Human Resources noted that administrators rely heavily on IRSs to identify problems, in spite of the well-known underreporting problems [11].

Despite extensive studies on IRSs, few researchers have identified or investigated the different stages of incident reporting. Most studies tend to focus on the reporting phase, whether and how it occurs [6, 12–15]. Less attention has been given to what happens after a report is entered. Yet, studying what happens post-report submission is essential because it allows us to discover if IRSs contribute to or fall short of enhancing patient safety and to understand how this occurs. Further, few studies have attempted to compare IRSs in different organizations or departments. Studying more than one case allows researchers to see dynamics across cases, “to understand how they are qualified by local conditions, and thus to develop more sophisticated descriptions and more powerful explanations” ([16], p. 172).

The purpose of this study is to understand the different stages of electronic incident reporting and to do so in a comparative study of two hospital divisions: General Internal Medicine (GIM), Obstetrics and Neonatology (OBS/NEO – for the purposes of this study, Obstetrics and Neonatology will be treated as a single division except where noted). These two divisions were chosen because they used the same electronic IRS differently. In addition, OBS/NEO was one of the earliest divisions to engage in electronic incident reporting, while GIM adopted the system later. Our preliminary discussions with divisional representatives had previously indicated that the dynamics associated with the IRS were different in the two divisions, and we thus pursued an in-depth study of these two cases.

### Conceptual background

IRSs are complex socio-technical systems. Øvretveit provided the introduction to a special issue of Social Science & Medicine, stating that

The social sciences are increasingly viewed by policy-makers and implementers as a resource for helping with the considerable challenges they have encountered in ‘implementing’ changes which are thought to be necessary to improve safety and quality…the value of social science perspectives [include] questioning common assumptions and showing why some strategies–such as voluntary incident reporting – are not meeting their aims ([17], p. 1780,1782).

Hor et al. found that there is a multiplicity of accountability roles affiliated with incident reporting, stating that “the incident reporting system and its policy are interwoven with other accountabilities in the local context” and that “local accountabilities can also be in conflict with the aims of the incident reporting system and the incident management policy” ([18], p. 1097).

**Fig. 1:**
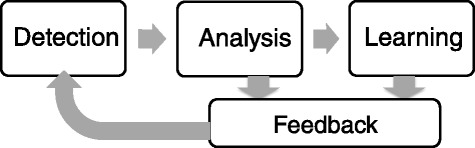
A high level depiction of an incident reporting system

Incident reporting is fundamentally a multi-stage process [19, 20], as shown in Fig. 1.

Generally, information enters the IRS at the detection stage, the reports are investigated and analyzed at the analysis stage, which feeds into the learning stage, from which incident reporting leads to some change in understanding or practice. Feedback can occur at the analysis and/or learning stages.

#### Detection stage

The majority of the studies of IRSs to date have focussed on the first stage; reporting. A few studies have described the reporting phase: Tighe, Woloshynowych, Brown, Wears, & Vincent [21] reported that a nurse or physician filled out a paper form with the aid of an incident book, classifying the type of incident in broad, pre-defined categories with as much detail as possible, up to including contact detail of witnesses; Cunningham & Geller [22], noted that the reporter was given reporting criteria and a form with check boxes and free text in which to enter an event description. However, the majority of studies focussing on the reporting of events into IRSs highlight the factors enabling or inhibiting reporting. Barriers that have been identified to prevent detection and reporting of incidents are numerous, and include fear of exposing incompetence or reprisal (both public and medical), lack of time, lack of education on what is a reportable incident, lack of feedback and futility [23–25]. Studies identifying enablers of detection and reporting are less numerous, and include incident severity, evidence that the profession values reporting, greater availability of reporting pathways, timely feedback and visible changes linked to reports in the IRS [13, 24, 25].

However, not all studies of the first stage of reporting identify enablers and inhibitors to reporting incidents. For example, Waring [26] proposed that the emphasis on assigning blame in incident reporting neglects the culture of medicine, such as physicians’ belief in the inevitability of error and viewing reporting as a bureaucratic exercise. Hewitt et al. [13] looked at the frames underlying nurses’ and physicians’ decision to report, and found that attention is rarely given to systemic, larger organizational safety issues. An underlying message of a variety of studies [15, 27, 28], is the need to increase the number of reports entered into the reporting system, and as such, these studies remain focussed on the first stage of reporting.

#### Analysis stage

Fewer studies have looked at other aspects of reporting such as the analysis stage. Pham, Girard, & Pronovost [29] recommended investigating reports thoroughly and involving multiple stakeholder input to enhance the value of IRSs. The large quantity of reports entered into IRSs has been noted as a possible barrier which can limit the ability to do meaningful data analysis [30, 31]. Bush [32], in a descriptive study, traced how a reported incident was investigated, describing how a multi-disciplinary team interviewed those involved in each incident, and subsequently met to discuss and agree on findings. Tighe et al. [21] described a clinical risk management team which collected completed reporting forms, assigned severity and likelihood of recurrence scores, and then entered the information into a central reporting system. Cunningham & Geller [22] described a review process whereby individual managers wrote their follow up action on the same reporting form filled out by the reporter, but in a different text box. In Waring & Currie’s [19] study of a UK hospital, reports from the hospital were analyzed by a central risk management department, sometimes with a brief assessment which prioritized managerial accounting over the contextual narrative describing the incident, overriding the reporter’s effort to provide all relevant details to understand the event. As these studies show, there are a number of different ways in which reported incidents can be analyzed, but only a few studies of IRSs describe these processes, and even fewer studies undertake a comparative study of incident analysis.

#### Learning stage

Some studies have addressed learning in hospital settings. Bush [32] described how an interdisciplinary team that investigated incidents then presented their findings to a Quality Assurance Committee with senior leadership, whereby the recommendations were oriented to system fixes (changes in design) as opposed to individual fixes (training). Tighe et al. [21] described how, once the clinical risk management team entered reports into the system, the same team reviewed the reports monthly, and followed up investigations and/or actions. In Cunningham & Geller’s [22] study, managers who filled out their section of the reporting form collected the reports and sent them to a central risk management department for review and database entry. Generally, however, IRSs are seldom cited as the genesis of learning interventions. Mahajan [33], focusing on the IRS, stated that the current paradigm of quick judgements and assigning of blame does not promote learning, whereas analysis with a human factors lens and then feedback to the reporter are key practices promoting learning from IRSs. The few studies that describe the learning phase in IRSs range from storing reports to interprofessional meetings with an accountable process to follow up corrective actions, yet comparisons between different IRS learning processes are not present in the IRS literature.

#### Feedback

In the World Health Organization’s (WHO’s) guideline on how to establish reporting systems, feedback is emphasized as a key feature. “Even with simple systems that focus primarily on recognizing hazards, resources should be available to support follow-up on reports, provide feedback to the reporter, and conduct at least a limited investigation when indicated” ([4], p. 55). In a study of the UK NHS, fifteen different aspects of feedback were highlighted as recommendations for IRSs [34]. Overall, if data is collected, including that from IRSs, it serves little purpose if its effects are not fed back to the reporter [35]. Feedback is an important yet often overlooked area of IRSs.

#### IRS in GIM and OBS/NEO

Incident reporting has also been studied in specific hospital departments. As this present study investigates incident reporting in the division of General Internal Medicine (GIM – a subset of the Department of Medicine) and the divisions of Obstetrics and Neonatology (OBS/NEO - a subset of the Department of Obstetrics/Gynecology & Newborn Care), a brief review of the literature concerning incident reporting and patient safety in these two divisions is warranted. General internal medicine is a core hospital division, and takes care of a wide variety of patients and patient conditions, although the vast majority of the inpatients are elderly. A number of studies of IRS use in GIM have been conducted. One study, aiming to improve reporting rates by reminding residents to report, found that the programme only succeeded in the short term [36]. Another study used Root Cause Analysis (RCA) in the analysis of reports, and revealed that human error is often linked to technical and organizational causes [37]. Obstetrics and Neonatology’s patients are pregnant mothers and at risk and premature babies respectively. A study found that the overall perception of safety and management support predicted reporting behaviour in a Neonatal Intensive Care Unit (NICU) [38], while positive team dynamics in labour and delivery were found to decrease the need for incident reports [39]. Another study recommended a structure for a critical IRS in Obstetrics and Neonatology, suggesting specific incident categories and a detailed review process [40].

However, despite the richness of the literature on individual departments and their struggles at the reporting stage, few researchers have analyzed departments in parallel in a comparative study.

This qualitative comparative case study of GIM and OBS/NEO hospital divisions attempts to fill some of these gaps by answering the following research questions:

*Research Question 1: What are the similarities and differences in incident detection, the analysis process, and learnings in two hospital divisions, GIM and OBS/NEO?*

*Research Question 2: What factors account for these differences?*

This study extends work that has been done on IRSs by going beyond the reporting stage and by undertaking a comparison of the IRS processes in two departments.

## Methods

Qualitative studies are useful for inquiries that ask what, how and why questions, “which help us to understand social phenomena in natural (rather than experimental) settings, giving due emphasis to the meanings, experiences, and views of all the participants” ([41], p. 42). As the present study seeks to understand the workings of an IRS in different settings, it is suited to a qualitative research approach. The study adopts a comparative case study design. Comparative case studies help researchers avoid jumping to conclusions with limited data, avoid ignoring disconfirming evidence, and prevent them from being overly influenced by higher profile study subjects ([42], p. 540). A comparative case study design is more robust than studying a single case, as replication can be realized – either literally (when similar results are obtained between cases) or theoretically (when contrasting results between cases emerge) [43].

The study focused on voluntary incident reporting and patient safety in a multi-campus teaching hospital in Ontario, Canada. The IRS at the hospital was available to employees through any networked device. The general process involved the following: The reporter entered information using the patient’s medical record number, identified the event, and provided a narrative describing the patient safety incident using facts. Incident investigators at the hospital were informed of an incident report by email and could forward reports to other departments for further investigation. Once reports were investigated and considered closed (and removed from email), they were forwarded to Core review for larger hospital issue investigation and archiving.

Data collection began in spring 2012 in GIM and ended in fall 2014 with OBS/NEO. Our data collection in each department started with our attending a quality review meeting where the researchers were introduced to key personnel who would later become interviewees. These key individuals helped us access other interviewees by contacting managers and practitioners. Over five months, two researchers (both independently and together) confidentially interviewed GIM participants; a similar process was adopted for OBS/NEO. Overall eighty-five participants were interviewed as shown in Table 1.

**Table 1:** Generic interviewee titles

Job Category GIM		Job Category OBS/NEO	
Physicians	11	Physicians	8
Nurse Leaders^a^	5	Nurse Leaders	15
Bedside Nurses	15	Bedside Nurses	15
Pharmacy	3	Midwives	3
Physiotherapists	3	Respiratory Therapy	4
Nursing Support	3		
Total	40	Total	45

^a^Nurse Leaders is a tem referring to all nurses with a job function not exclusively at the bedside

The interview included questions about the IRS: how it was introduced, structured and used. There were also questions about safety practices and safety culture. Interviews averaged approximately 45 min, and were digitally recorded and subsequently transcribed. Data analysis was undertaken by two researchers who met to discuss the themes in the interviews and the derivation of codes. Atlas ti software (GmbH, Berlin, Germany) was used to code the interviews and retrieve quotations. The analysis involved both a deductive and inductive approach [16]. Through a reading of the literature, we were informed about concepts and approaches related to IRS and patient safety (deductive approach); some sample codes based on the literature included Fear of Reporting, Feedback, Individual Staff Focus and Systems Thinking. Our analysis of the data revealed local practices related to the use of the IRS (inductive approach); some sample codes derived inductively included Pre Screen, Quality Assurance (QA) committee, Litigation and History. Through an iterative process of moving between the literature and the data, we identified differences in reporting, analysis and learning in GIM and OBS/NEO, as well as the reasons why these differences exist. We conducted the analysis for each department separately and then engaged in comparison. In other words, we followed Miles and Huberman’s [16] recommendation to do a within case analysis followed by a cross-case analysis.

This study underwent ethics review at both the hospital where the study was conducted, and the researchers’ university. Trustworthiness [44] was established by ensuring the researchers were in constant communication, questioning potential biases and assumptions, and returning to the data when there were disagreements. Member checking [44] was undertaken through presentations to interprofessional (quality) meetings of the different divisions and seeking feedback on the results reported.

## Results

In this section, the divisions’ experience with incident reporting is analyzed: first, GIM, then OBS/NEO. In each division, the reporting process is examined through reporting events, analyzing events, learning from reported events and system feedback.

### Detection, analysis, learning and feedback in GIM

#### Incident detection – GIM – predominantly nurses, outcome based

Incident detection in GIM was examined in its connection to the history of incident reporting in that division. Historically, incident reporting was on paper, done exclusively by nurses, and was seen as punitive. The fact that it was a paper based system tended to restrict its use to nurses: “the whole sort of paper incident report used to be largely just nursing generated” (*GIM Physician 6*). Although physicians and other practitioners could and did report into the present IRS, they did so much less than did the nurses.

I think we’re just so used to thinking of ourselves as a unit in terms of nursing practices and nursing processes and we’re so used to dealing with issues within our own scope of practice that I don’t think many people think of [reporting] as being a tool for physician improvement as well. (*GIM Nurse Leader 4*)

Furthermore, there was a perceived punitive component to this reporting. The perception of the old system being blame-based lingered in the minds of some of the nurses with longer tenures.

For anybody that’s been here as long as I have, you had 3 medication errors and you were being disciplined…I don’t know if that was even indeed the case but that was what I grew up being a nurse at the bedside being petrified of ‘oh my goodness if I made this error’ (*GIM Nurse Leader 3*)

However, at the time of the interview, interviewees stated their knowledge that the IRS was non-punitive, and that the intent was to learn from reported events. A nurse described, “Med errors are not obviously in favour of my career …but incident reports … should be looked at so [incidents] can be stopped in whatever way possible” *(GIM Bedside Nurse 11).* Many nurses espoused similar opinions that the present IRS was non-punitive. Physicians were more skeptical about the IRS being non-punitive.

I think there’s still quite a culture that people are afraid to report things because of either sort of punishment in the future whether it be medico-legal punishment, punishment from a colleague or a superior or causing a relationship to deteriorate between 2 staff physicians because someone told on me basically. *(GIM Physician 7)*

With nurses more or less believing that the system was non-punitive, what did they report? Overall, falls and medication errors comprised the vast majority of reported incidents in GIM: “The things that come to us most frequently are things that are nursing related; medications, transcriptions, falls” *(GIM Nurse Leader 5).* These incidents were realized – a patient had fallen, a medication error had occurred. The orientation of most of the reports was outcome based, that is, the outcome determined whether or not a report should be written. This had consequences for near miss reporting, as by definition near misses do not have a negative outcome. Hence, near misses were rarely reported, despite the corporate messaging that they should have been.

Near misses…I think people are thinking it’s not an incident, it’s a near miss, even though we should still report them. It is still time consuming so I think near misses don’t get reported as much as they should, if at all. *(GIM Bedside Nurse 3)*

In summary, in GIM, typical incidents were outcome based (chiefly falls and medication errors), and reported principally by nurses. Despite the blame-based past, nurses stated that reporting was now generally non-punitive.

#### Analysis process – GIM –siloed approach

The unit level review was undertaken by a nurse leader who, upon reading the incident in email, decided on the level of follow up. The follow up that the nurse leader engaged in directly was often focussed on an individual that was involved with the reported incident. An example on narcotic disposal is described below, along with the intervention that the nurse leader did with respect to this incident.

There is a proper way of wasting a narcotic and [the nurse] didn’t use that. So that would be my recommendation and then details of follow up would be: [The nurse] needs to review the narcotic [policy]. *(GIM Nurse Leader 3)*

In some situations, the individual described in the report (with whom the follow up would be conducted) was unknown. “With this [IRS] I don’t have the assignment readily available; I don’t have the chart readily available so I can’t make the investigation to see who the nurse was. So my solution is to present it at a staff meeting, but again it doesn’t make it as meaningful” *(GIM Nurse Leader 3).* In contrast to an individually focussed approach, the nurse leader might have viewed the incident with a systems lens, seeing the incident in a larger context. A nurse leader reflected on systems approaches, instead of focusing exclusively on an individual: “Because if you made a mistake, most likely it’s human error or there’s some system in place that was just not working” *(GIM Nurse Leader 2).* However, a systems view was not as frequently engaged with as the individual view.

Once the investigation was over, the nurse leader closed the incident report. The incident reports were also separately reviewed by a physician (clinical reviewer) to determine if harm had occurred to the patient, and if it was avoidable. The sequence of the nurse leader review and the physician review was not clear – they may have been in an order, or simultaneous. “You write out what it was and then there’s a check…‘was that related to the medical treatment or was that related to the medical condition?’ and ‘was it preventable?’” *(GIM Physician 2).* The level of information given, as the majority of reports were written by nurses, was often insufficient for the physician reviewers to undertake a full review.

Well the nurse for example doesn’t go into the detail that you would like to have into the case…. [A review] took me almost an hour in just trying to figure out what that person was trying to say what happened…When a physician reports, especially if we’re dealing with a more medical issue, it’s a little bit better to be done by somebody who has a little bit more knowledge into the medical issues. *(GIM Physician 3)*

Some users stated that the system had yet to advance beyond data collection, implying that the potential of the IRS had not been realized. “We need to be sitting down probably with the nurse leaders or somebody from the Division, looking at how you prevent medical errors… The way I perceive it [the IRS] is just data collecting at the moment” *(GIM Physician 2).*

All in all, in General Internal Medicine the various reviews took place independently – nurse leaders and physician reviewers often did their reviews in their offices, reviewing the same (typically nurse) reported events. There was some follow up at the individual level for nurses but there was no joint (physician and nurse) overview of reported incidents.

#### Learning through reporting – GIM – ambiguous linkage to reports

Nurse leaders stated their view that the learning that individual nurses received were “teachable moments”, where the approach was non-threatening, and the individual felt safe to discuss incidents with the leader.

I always try to use it as something like a teachable moment … You don’t want people to be afraid to tell you they’ve made a mistake and so I think we’ve done a very good job. But people still are very nervous… All we want to do is learn from this …as long as you walk out with a way to improve your practice that’s what I believe it’s all about, to make it safer for the patients. (*GIM Nurse Leader 2*)

A systems view could also result in learning from reported incidents,

For me it’s very helpful because now I can see trends… [People] individually have their own problem, but this now allows us to see it as a systems issue. So we notice that this mistake is happening with this medication or this process so we can go back and discuss it. We are able to pinpoint a systems issue rather than reflect on one individual issue, which for me is very helpful because it’s education, it’s global, it’s not a problem with a nurse, it’s usually related to a system. *(GIM Nurse Leader 4)*

This approach was not as common as individual “teachable moments”. When asked what learning emerged from the IRS, a physician reviewer noted flagging cases suitable for Mortality and Morbidity (M&M) rounds.

From our point as the Clinical Reviewers we review them all and then we will note which ones we think might be important to review within the Division as far as for Mortality & Morbidity rounds. So things of more clinical interest instead of structural or administrative changes that need to be looked at. *(GIM Physician 2)*

M&M rounds were meetings that traditionally physicians attended, to analyze a case in detail to determine if there was, as a physician stated, a “cognitive” (a decision error on the part of the physician in the case) or a “system error”. However, since physicians did not report frequently, many of the M&M cases had to be obtained through personal communication: “We haven’t been using the [IRS] as our database to gather the cases” *(GIM Physician 4)*. Given that these rounds were for physician learning from cases, they didn’t have an interdisciplinary audience. Overall, nurses’ learning was mostly through individual “teachable moments”, and less effort was exerted on systemic issues that might have been identified through the IRS. M&M rounds were to allow physicians to discuss catastrophic cases, which may or may not have been informed by a report in the IRS.

#### Feedback – GIM – weak

Some of the participants did not know what happened to reports they wrote. “We hit a send button and I never hear about it again. It does nothing for me.” *(GIM Bedside Nurse 1)*. Others had a vague idea of the review that happened, “My manager will sometimes follow up with me. But whoever else it goes to, these people who review it, perhaps it goes to researchers, I don’t know” *(GIM Bedside Nurse 8).* While staff meetings may have given back some information to reporters, not informing the reporter on the change that their report prompted got the reporters to believe their time was not valued: “I’m taking 10 min of my time [to report] I’d like to know that it’s at least helping… They would probably encourage us to do more if we see ‘oh it’s making a difference’” *(GIM Bedside Nurse 6).*

It was also possible for reporters to become cynical of the IRS due to lack of feedback, which could have a demoralizing effect and serve as reason to not use the IRS.

Honestly it was an event that very significantly affected me emotionally but I didn’t hear anything back from it. I didn’t get any feedback as to how this was rectified and how we’re gonna change the system or anything really. And so I think that was really frustrating and that’s probably why I haven’t been motivated to use it again. *(GIM Physician 10)*

Overall, feedback based on the IRS was identified as a major weakness in GIM.

#### Summary – incident reporting, analysis, learning through reporting and feedback – GIM

Incident reporting was done primarily by nurses. The types of incidents recorded were primarily outcome based, and included mainly falls and medication errors. The analysis was undertaken by individual nurse leaders who more often than not had a focus on the individual. The individual physician reviewers assessed if harm was preventable, sometimes with difficulty. Learning through reporting mentioned by interviewees was individual “teachable moments” for nurses delivered by nurse leaders, and physician reviewers identifying cases for M&M rounds, although most M&M round cases were not informed by the IRS. Divisional knowledge of the incident analysis process was limited, and feedback to the reporters (aside from staff meetings) was rare.

### Detection, analysis, learning and feedback in OBS/NEO

#### Incident detection – OBS/NEO –team approach, near miss reporting

In this section, we grouped Obstetrics (OBS) and Neonatology (NEO), but there were some differences between the two departments in practices, which we indicate where pertinent. In the past, OBS had an IRS unique to them, prior to the current version of the organization-wide reporting system. It was developed in house, and was not accessible beyond this division. However, many reported that the specific OBS IRS facilitated the transition to the present IRS in OBS. Below, nurse leaders described the history with the OBS specific IRS, and how it provided a background for the present day reporting practices.

[The OBS IRS] was exactly what the [current reporting] system is all about. And we were doing it years before the [current IRS] was invented. So I think most of the people in the Birthing Unit are quite comfortable on reporting the cases because we’re reporting the same things. (*OBS/NEO Nurse Leader 12)*

It was the very same philosophy [as the current IRS]; near misses, misses, policies that weren’t being followed. (*OBS/NEO Nurse Leader 7)*

OBS customized the present IRS by creating a drop down menu of specific indicators – beyond only the free text box that guided practitioners on what to report – inspired by experience with their OBS specific system. Near misses were expected in the IRS. Near misses could be general (e.g. about to give the incorrect medication) or specific drop downs (e.g. a newborn having pH of gases <7 or >12.5), and were consistently reported, as can be seen from the quotes below.

I think the incident reporting system is probably good for [being proactive] because if it’s a near miss then that can indicate an issue that needs attention before it’s an outcome that’s not a near miss, a definite incident where someone was hurt. *(OBS/NEO Bedside Nurse 2)*

[We report bad outcomes and near misses] because they’re both learning experiences *(OBS/NEO Bedside Nurse 4)*

Drop down menus helped facilitate near miss reporting, and there was a clear expectation and a willingness to report:

They’re gonna be reluctant to put things in say[ing] “Why are you [reporting] that?” “Oh because the PH was 6.9 and anything <7 we have to put it in” and the person says “Okay even when the baby’s fine?” “Well even though the baby’s fine it just has to go in” so I think that kind of defrays some of the why are you putting that in. *(OBS/NEO Physician 3)*

I just fit it in my day. It’s like everything else I do it’s just part of the duties it’s just an accepted part. It’s not an exception, it’s not a burden, it’s just part of the duties. *(OBS/NEO Respiratory Therapist 2)*

Near miss reporting was a well-established practice in Obstetrics, with interviewees having stated near miss reporting was part of their reporting culture.

There appeared to be no blame in incident reporting in Obstetrics. Further, emphasis on an interdisciplinary team approach ensuring completeness of the incident report was the focus.

On this unit we have a very close rapport with the doctor …we have to be a tight unit because when the emergencies are happening we need help and we usually work as a tight unit. So if something goes wrong we talk about it and we make sure that all the information is filled in for it to not happen again and I don’t feel like people are trying to blame each other when we fill these, it’s just to improve how things are supposed to run. *(OBS/NEO Bedside Nurse 5)*

We have moved away from the culture of blame to more of a team-based culture. *(OBS/NEO Physician 3)*

Overall, Obstetrics had a history of incident reporting, and reporting near misses was a well-established practice. Practitioners generally indicated that blame was not a concern, and that an interprofessional team approach was important.

#### Analysis process – OBS/NEO – multidisciplinary team approach

Historically, the OBS IRS “generated a very similar review process to what the current [IRS] process has” *(OBS/NEO Nurse Leader 4).* As such, there was more experience and familiarity with the review process in Obstetrics. Incident review followed a sequence starting with a designated nurse leader or a delegate.

You have to go through all the documentation … Sometimes you do need to interview the physicians and/or nurses to find out because the documentation isn’t clear … … I try not [to delegate] cases that are too complex …. *(OBS/NEO Nurse Leader 10)*

This review involved an investigation, and may have had an individual focus and the report closed at that point. However, most cases were reviewed both at the nursing level and at pre-screen.

The pre-screen phase had a multi-disciplinary team looking at the completed reviews monthly: “[An obstetrician], the manager of the clinics as well as the critical care leader from Labour & Delivery and then often one of the care facilitators, so the 4 of them go through each case” *(OBS/NEO Nurse Leader 5).* The pre-screen committee did an analysis of its own.

Was there harm? Was there potential for harm? Was there no harm? Was there no potential for harm? From a 4 to a 10 you have to rate where you feel there was definitely harm. Was it because of medical care? Was it likely not because of medical care?… So in order to close an [incident report] and send it to the archives you had to answer those questions *(OBS/NEO Nurse Leader 5)*

The pre-screen committee decided on what to archive, what to follow up with further at the unit level, and what to bring to the Quality Assurance (QA) committee. The QA committee, which met monthly, was a multidisciplinary departmental committee with representation from all the divisions (including neonatology), and all the professions involved in OBS and newborn care. The types of cases that were brought forward to QA were those that had some system learning potential.

What goes to the QA table mostly is systemic problems. So if, for example, there’s a communication breakdown between Anaesthesia or Neonates or whatever that goes to the QA table every single time because that can be prevented. *(OBS/NEO Nurse Leader 2)*

The analysis in QA involved a discussion inviting all at the table to contribute their professional opinions.

Everybody is pretty forthcoming and they hash it out at the table and you get a different perspective put on it and yes that makes sense, right. It takes some of the reaction out of it, when you get the different approaches, it’s not all about me, you can see the other sides to different things *(OBS/NEO Nurse Leader 11)*

The QA committee was seen positively by most who were involved in the QA process.

Out of all the different obstetrical units I’ve participated in I think that the way they’ve developed the QA meetings and persistence with the QA meetings and the way it’s recorded, reported and followed up is probably the most impressive… They have gone a long way to try and promote patient care and safety that way. *(OBS/NEO Physician 4)*

Overall, with the emphasis on multidisciplinary team meetings to review incidents at multiple levels, incident analysis in OBS/NEO was an inclusive process that allowed each discipline’s voice to be heard.

#### Learning through reporting – OBS/NEO- strong linkage to reports

Often changes took place because of reported incidents; for example, a nurse talked about a call bell system problem that got resolved soon after he/she and others reported it, “So that was very positive. We felt like oh these people are listening to us. We’re not just filling these for nothing so that’s good” *(OBS/NEO Bedside Nurse 5)*. Other experiences of linking changes to incident reports were mentioned such as a rewrite of an HIV medication order sheet and a repair of an anesthesia call button in the OR. However, one intervention that was prompted by incident reports was spontaneously spoken of by multiple interviewees in Neonatology.

*(OBS/NEO Nurse Leader 4)*: …like unplanned extubations where they’re supposed to complete an [incident report], we just said as a way of tracking, because sometimes it’s only when you look at something that you see there’s a pattern. Because it might happen to her, it might happen to her, but it doesn’t happen to the same person and so people don’t think about it.

*(Interviewer):* And that was found through [the IRS]?

*(OBS/NEO Nurse Leader 4):* Yeah, yeah.

*(Interviewer):* Do you know of any changes that have come about because of the [IRS]?

*(OBS/NEO Bedside Nurse 13)* Definitely like the accidental extubations is primarily one that we are learning from absolutely because it’s only through that that we’re learning how many are happening and…from that we can identify that more babies are being self-extubated than we would really like to see basically.

A quality improvement initiative for unplanned extubations was spearheaded by a respiratory therapist, who was asked to begin noting how many accidental extubations were actually happening.

So I put a memo and I say, okay we need to really track this guys, we think it’s a problem. We need to have it reported. So we kind of made them alert … and defined what’s an accidental extubation… *(OBS/NEO Respiratory Therapist 1)*

These accidental extubations were not frequent to an individual practitioner – only when looked at collectively would anyone know there was a problem, which could be done through the IRS. Instead of only reminding individual practitioners to be more careful, the approach was to see these accidental extubations as a systemic problem. When the respiratory therapist learned that other neonatology departments were having similar problems, it lent credence to the idea that it might be a problem at this hospital too.

I went to a conference and [another hospital] presented accidental extubation so it was an issue for them too…We had a long phone interview with a physician at University of Pennsylvania [They were working on the same problem] *(OBS/NEO Respiratory Therapist 1)*

The work was a quality assurance project, with support from the corporate quality department (unrelated to the QA committee in OBS/NEO) and training sessions and roll out were done with their help. At the time of the interview, the project seemed to be a success.

We’re now at 135 days today and when we tracked and looked…We went 133 days between the last extubation to the next accidental extubation, the longest stretch we ever did…so now we’ve passed it…*(OBS/NEO Respiratory Therapist 1)*

#### Feedback - OBS/NEO – moderate

Notwithstanding the feedback of having a reported problem fixed (the best feedback possible), informing the reporter was still challenging. Staff meetings were a way of disseminating information, and other feedback also occurred occasionally, especially if the reporter was invited to share his/her viewpoint at a QA meeting. Additionally, there was a bi-annual newsletter that went to the department (including OBS/NEO) called “Closing the Loop”, but due to its general dissemination, individual reporters mentioned appreciating receiving feedback: “I know there’s that Closing the Loop Newsletter that goes around but it’s very general… As far as us reporting our own thing I don’t think it’s very specific about that… “*(OBS/NEO Bedside Nurse 2)*

Those in positions who could initiate more feedback to reporters realized this weakness of the system, and had plans to improve this aspect of incident reporting. “Something I think we could do better at is closing the loop with staff. So [we need to] figure out how to do that and that’s something that I’d like to work on over the next year to do that a little bit better.” *(OBS/NEO Nurse Leader 9).* A physician stated, “if they took the time to put the information in [the IRS], somebody [should then] take the time to give the information back” *(OBS/NEO Physician 4).* Most participants believed that there was room for improving feedback to reporters.

#### Summary - incident reporting, analysis, learning from reporting and feedback-OBS/NEO

OBS had some experience with a previous IRS, whereby non-punitive reporting including near misses was an ingrained practice. A team approach was common. Analysis with an individual focus was done by nurse leaders, who recommended reports with a system focus (most reports, according to the nurse leaders who first reviewed the reports) to the multidisciplinary pre-screen committee that reviewed incidents. Reports with systemic learning potential were often brought forth to the multi-disciplinary departmental QA committee, where they were reviewed and typically policy changes were made. Changes could be traced to the IRS. The department had a newsletter that highlighted the IRS, but individual feedback to reporters was a priority for those who could effect local change.

A summary of the differences in incident detection, analysis, learning and feedback between GIM and OBS/NEO is shown in Table 2.

**Table 2:** Comparison of the incident reporting process in GIM and OBS/NEO

Reporting Aspect	GIM	OBS/NEO
Incident detection	Predominantly nurses	Team approach
	Predominantly outcome based (falls and medication errors)	Outcome and near miss based
Analysis	Individual nurse leader investigation	Individual nurse leader investigation
	Individual physician review to determine preventable harm	Multidisciplinary pre-screen committee review
		Larger multidisciplinary QA review for policy type issues
Learning through reporting	Feedback to individual nurses through “teachable moments”	System focus
	Ambiguous linkage to incident reports (e.g. M&M rounds)	Clear linkage to incident reports (e.g. Accidental extubation project in NICU)
Feedback	Staff meetings (nursing)	Staff meetings, bi-annual newsletter, occasional reporter participation in QA meeting

### Factors that influence the IRS practices in GIM and OBS/NEO

The main influences on IRS use were seemingly the threat of litigation, the introduction of the IRS and interprofessional training. In what follows, we compare how each of these influences affected incident reporting practices in the two departments.

#### Influence of litigation

In GIM, physicians spoke more than nurses about the legal influence on their overall practice, and their reporting practices in particular, indicating that the threat of a lawsuit was one of the barriers to physician reporting.

That might end up in the courts if you get [a] litigious family … you can always get a lawyer who will make something of it. So [doctors] tend to then failsafe… there is a certain amount of defensive medicine …*(GIM Physician 5)*

[Doctors] aren’t really sure what’s gonna happen to [reported] information and I don’t know if they’re worried if it’s a medical/legal issue…*(GIM Physician 4)*

The threat of litigation influenced physicians’ practices in GIM as there was concern that reported information could lead to legal proceedings. However, the threat of litigation was stronger in OBS/NEO.

In the adult world [the window of litigation] is 3 years. So I could have something go wrong in that world and outlive the issue. But in the neonatal world …our window of litigation is age of majority plus 2 years… it can come back to haunt you in 5 years, 7 years, 10 years and if you have no record of it, now what? *(OBS/NEO Respiratory Therapist 2)*

The statute of limitations, or how long a complainant has to launch a lawsuit, was far longer in OBS/NEO than in GIM. This was one of the factors that influenced reporting practices. If there was no record of an event, and a lawsuit was launched, the practitioner would be in a difficult position. Furthermore, the amount of money in the OBS/NEO lawsuits was substantial.

Our specialty is the most litigious specialty …Of the Canadian Medical Protective Association [payouts], close to about 28 % [are from] OBS/Newborn Care mostly OBS … When the cases do go bad, the outcome is a lifetime of disability. That’s why our staff here has embraced this system-related issue for the risk management far earlier, far more aggressively than some others (*OBS/NEO Physician 5*)

In contrast to GIM where incident reports could be seen as a trigger to lawsuits, physicians in OBS/NEO expressed a different view.

The lawyers aren’t gonna go after you just because you’ve had an incident report. The lawyers are gonna go after you because the patients have started the process because something bad has happened. So better that we do the incident reporting so bad things don’t happen, I think is the kind of way that we look at it. (*OBS/NEO Physician 7)*

The threat of litigation is seemingly proportional to the use of the reporting system – the lower the threat, the less the reporting system is used (GIM), and the higher the threat and the longer the period of potential threat, the more the reporting system is used (OBS/NEO).

#### Introduction of the IRS

The way the IRS was introduced to leaders and to the front line staff in the two divisions was revealing. In GIM, a Nurse Leader was invited to help customize the system for use in the unit, but the initiative came from the IRS team.

How was it introduced? I absolutely remember. I got invited to a meeting that was 4 hours long and I had no clue what it was about, is how I was introduced to the whole thing. But then teaching came out after that where we were part of the pilot actually helping to formulate the actual form that’s online now. So they would ask us what we thought. What information needed to be in there? What would make it easier for the staff filling it out? *(GIM Nurse Leader 2)*

The corporate project team invited contributions from those whose divisions were going to be affected on how the project would look in their area. As such, while there may have been some customization of the IRS for GIM, it was at the invitation of the corporate project, although it is clear from the quotation that this contributor was not initially in the know regarding his/her role. The requirements for the customization of the IRS for GIM were discovered and incorporated after the division was invited to trial the IRS in a pilot project.

Another aspect of how the IRS was introduced involved training the front line staff. In GIM, this training focussed on the logistics of reporting – how to access the reporting form, what boxes to fill out and where to click to submit. This training was provided as part of the roll out, and was given by the corporate team.

I believe I just got a little in-service at the hospital…very quickly showing us the screens and what information needs to be inputted and that’s basically I think all that I remember really getting out of it. *(GIM Bedside Nurse 2)*

There might have been myself [and] just 3 or 4 people sitting around a little corner of a computer and she just showed us how to enter in an adverse event. *(GIM Bedside Nurse 12)*

(*Interviewer*)In your training that you had received when it was rolled out, was what should and should not be reported covered?

*(GIM Bedside Nurse 13)*I don’t recall that they had gone into saying what should and shouldn’t be reported.

Those on the unit who train the incoming nurses continue to train on the logistics of reporting

*(GIM Nurse Leader 5)*They actually [get] training in corporate. They also have to do an online module before they can have access to [the IRS] I do fill in the holes of showing them what screens to go to, how to work through the menu.

(*Interviewer*) In your training do you ever talk about what qualifies as an incident?

*(GIM Nurse Leader 5)* I don’t, no…[they] probably made an assumption that the [new nurses] would know what an incident is.

Overall, GIM was invited to a meeting by the corporate project team to customize their form, and the corporate and local training of users focussed on the mechanics of entering a report.

In contrast, OBS had their previous experience with their locally owned IRS, and was proactive in contacting the corporate project to ensure their known, specific needs were taken into account before the software came to their department.

So when the hospital started talking about a [corporate IRS] system I offered to us to pilot it because we had [the OBS IRS] and we wanted to influence what the new system was gonna look like and see what it could do for us. And originally the hospital wasn’t going to customize it. “It’s standard for everybody and it should fit” and we said “we can’t, it’s got to be tweaked in some areas. A lot of it can be standard but some of it needs to be tweaked”. (*OBS/NEO Nurse Leader 1*)

So we spoke with [the IRS] team in terms of putting into [the IRS] things that would usually be associated with adverse events in obstetrical cases. So these…we’ve classified them into system indicators or communication indicators or physician specific indicators and our broader classification would be a maternal or a fetal indicator. *(OBS/NEO Physician 1)*

Guided by the previous IRS OBS specific system, this local ownership continued in the way the present IRS was designed for OBS use.

Training also differed in OBS. Front line staff instruction focussed on more than only how to log a report.

We’re (1) trying to prevent something from happening again like a near miss or (2) helping to deal with certain situations better or how could we improve…how can we fix this in the future? And that’s the main thing that stood out when doing the training. *(OBS/NEO Bedside Nurse 4)*

This nurse began after the official roll out, and was trained on the unit.

When I came in my Educator [went] over what is an [incident] and what we should be completing [a report] for. *(OBS/NEO Bedside Nurse 6)*

While the detail of the reporting method is important, these interviewees did not mention that aspect, but did mention the reason to report. Obviously the mechanics of how to enter a report must be learned at some point, but the training in OBS focussed as well on the reasons to report.

The introduction of the IRS differed in GIM and OBS. The customization of the IRS for these two departments happened differently. In GIM, the process was initiated by the corporate project team in a top-down approach, and the customization of the IRS form came about through experience in a pilot project. The OBS division approached the corporate project with their pre-determined customization needs in a bottom-up fashion. The training also differed between the departments, with GIM focussing on the logistics of filling a report, and OBS going beyond this to cover the rationale for reporting.

#### Influence of interprofessional training

Various individuals in GIM were patient safety leaders, and individual practitioners had gone to conferences, and trainees (e.g. residents, nurses) were being increasingly exposed to patient safety in their residency and training, but there had not been a coordinated, systemic effort to educate GIM practitioners about IRSs. When looking at Neonate care separately from Obstetrics, this department shared the non-specific patient safety training experience with GIM –there seemed not to have been a deliberate effort to train the division. However, the issue was different in OBS.

Due to the threat of costly litigation, the Society of Obstetricians and Gynecologists of Canada played a key role in training labour and delivery professionals in patient safety, and this organization developed a course in 2002 called Managing Obstetrical Risk Effectively, or MORE OB, based on knowledge and procedures in the aviation industry. (MORE OB is now owned by Salus, a private company.)

So the brilliant thing with MORE OB is its standard obstetrical content that any care provider, nurse or physician can review and access; it’s a pre-test and post-test for knowledge… They picked all the high error and litigation issues that are common in Obstetrics; shoulder dystocia, post-partum haemorrhage, to give you examples and they created tools. (*OBS/NEO Nurse Leader 1*)

But the content of MORE OB was not the only “brilliant” piece. There was a major portion on interprofessional collaboration. It provided “safe spaces” for differing opinions:

And everybody has a right to ask a question and it is encouraged and fostered and learning beside each other. We shared each other’s viewpoints on how we cared and how we saw a case. And that was really good because both team members be it medicine or nursing commented about how good it was to have that sharing going on (*OBS/NEO Nurse Leader 7)*

MORE OB introduced multidisciplinary simulations to the obstetrical teams, which formed the basis of present-day simulations. It also had a substantial component on tackling the hospital hierarchy; during the didactic training sessions, nurses and physicians had the opportunity to teach each other, and everyone got the same message at the same time:

That was a wonderful program. It was phenomenal in the teaching aspect of it, it was great in the interdisciplinary aspect of it as getting us to all work together as a team. It was great to show the obstetricians our role in it all, and for us to see the obstetricians’ role. So I loved it. (*OBS/NEO Bedside Nurse 1)*

MORE OB also provided the participants with specific communication tools to help flatten the hierarchy in daily work, as expressed by physicians and nurses.

We’ve also empowered the nursing colleagues as well as the whole allied healthcare colleagues to go directly to the most responsible staff [physician] or designate…In the past regimented nursing model, they would just communicate within themselves and then one person would communicate. (*OBS/NEO Physician 5)*

We’re supposed to contact the junior resident first and then the senior resident and then the staff. If it’s a big emergency, I don’t even bother with the junior resident … I go straight for the senior and if I don’t like the way they’re treating the case, well I go to staff. (*OBS/NEO Bedside Nurse 5)*

The MORE OB training helped establish a culture of patient safety, as a physician stated,

“I think with a change in culture with the MORE OB we became a bit more proactive” (*OBS/NEO Physician 2).* This was echoed by a midwife.

Blameless, everybody has a voice, everybody has a job to report, and everybody has a role in this discussion. It’s in camera, it doesn’t go out the door other than positive things as to what we could change. It’s not finding blame it’s everybody critically analysing something objectively in a collegial manner with the patient’s safety as the driving force. (*OBS/NEO Midwife 2)*

As mentioned, the programme was limited to labour and delivery – neonatology was not a participating division in the training. However, some of the tools of MORE OB were part of the neonatology department too: “[The neonatologists] have told us several times, if you have an issue with a physician on nights give them a call 24/7” (*OBS/NEO Respiratory Therapist 3).*

Interprofessional training and culture were a substantial influence on reporting practices in OBS/NEO.

Table 3 summarizes the main influences on how the IRS was used in the two divisions.

**Table 3:** Comparison of the influences on the IRS process in GIM and OBS/NEO

Influence on IRS use	GIM	OBS/NEO
Threat of litigation	Moderate; had an inhibiting effect on, especially, physician reporting	Very high; had encouraged the domain to be proactive, and embrace IRS use
Introduction of IRS	Top down, customization asked of GIM	Bottom up, customization asked by OBS
	Instrumentally focussed training – how to report	Training focused on reasons to report
Interprofessional training	Minimal interprofessional training	MORE OB (in OBS division), which gave rise to several safety practices including the OBS specific predecessor of the current IRS

**Fig. 2:**
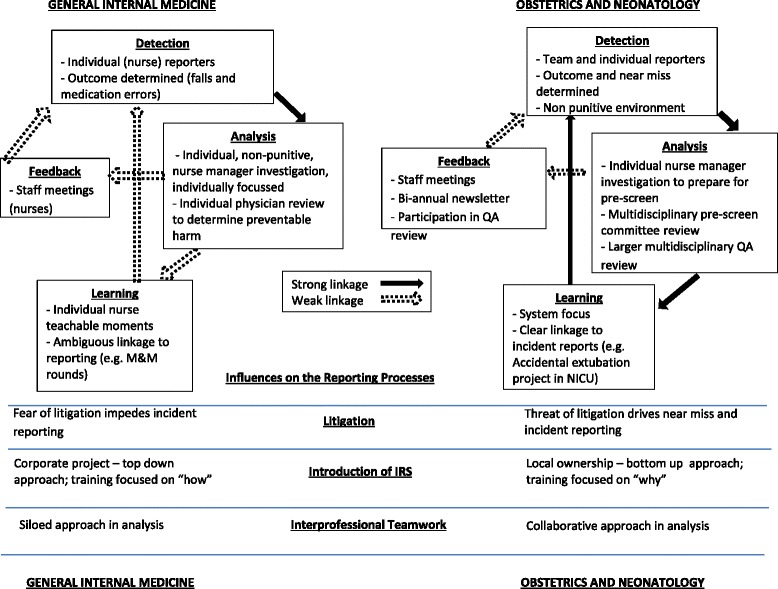
A descriptive model of the incident reporting processes in GIM and OBS/NEO, including influencing factors

A model of the use of the IRS and its corresponding influences is depicted in Fig. 2.

## Discussion

This study focussed on the differences in incident reporting, by examining incident detection, analysis, outcomes and feedback in two divisions: GIM and OBS/NEO. It also analyzed the factors that help to account for the differences in the two divisions. The importance of studying interventions to understand the underlying factors that enable success is growing in importance in patient safety generally [45–47]. This comparative study was undertaken in one hospital; hence the higher organizational level influences were common. Nonetheless, there were major differences in the approaches in the two divisions.

### The incident reporting system

In terms of detecting, the main reporters in GIM were nurses, and the main items reported were falls and medication errors. As discussed, these are outcome driven reports – the fall or the medication error happened, and that is the reason the report is entered into the IRS. In OBS, the reporters were of various professions, and the reported items were largely pre-determined on their locally-owned dropdown list, many being near miss situations. The near miss reporting has also been called process reporting, as the process triggers the report, not the outcome. The contrast between an outcome and process reporting system has been studied by Nuckols, Bell, Paddock, & Hilborne [48]. In studying the content of nearly 4000 reports from an academic and community hospital, they found that

In terms of their potential usefulness to improving patient safety, the process-oriented reports were far better for determining incidents’ preventability and identifying contributing system and provider factors. Outcome-oriented reports were better for identifying patient factors….Hospitals should specify which undesirable outcomes, if any, should be submitted to their voluntary incident reporting systems and should train staff to focus on reporting problems with care and describing those problems in detail ([48], pp. 143, 144).

When seen on this scale, GIM’s reporting is most similar to the outcome oriented reports and OBS/NEO is most similar to the process oriented reports, whereby Nuckols et al. clearly state that the process oriented reports have a greater potential to improve patient safety.

In GIM, nurse and physician analysis was undertaken independently – a siloed approach, similar to a previous study [22] described earlier. In Obstetrics, only the first stage of analysis was undertaken independently by a nurse leader, and all subsequent stages (pre-screen and QA committee) were undertaken interprofessionally. OBS/NEO had maintained a collegial environment, focussed on change and learning, similar to a process described earlier in a respiratory therapy setting [32] and an accident and emergency department [21]. Unlike the GIM model of individual analysis, the Obstetrics model of layers of interdisciplinary review allowed multiple voices to be heard, culminating in a department level QA interprofessional review.

Feedback in IRSs is a common problem generally, not only in healthcare [49, 50], and it acts as one of the more significant barriers to reporting in healthcare [23, 25]. However, OBS/NEO’s newsletter, an analysis process inclusive of the reporter (if the reporter chose to participate in the QA meeting), and most especially interventions linked to IRS reports have made progress on ensuring reporter’s voices were listened to. In GIM there had not been much progress to ensure the reporter’s experience was valued. Benn et al. [51] studied feedback in healthcare IRSs, and found a multitude of ways it was practiced. However, “Getting the content of feedback right in terms of the message it conveys regarding how incident data will be used, the level of anonymity provided to reporters and the potential consequences of disclosing errors and near misses through reporting are all critical issues that can impact upon reporting culture” ([51], p. 20). It is recommended that both GIM and OBS/NEO could improve their feedback processes, in order to ensure a more effective IRS.

The examples on learning from IRS reporting were also different between the divisions. In GIM, nurses often received the individual feedback on their performance through “teachable moments”, which may have led to individualized learning. Specific improvements based on the IRS data were not mentioned by interviewees in GIM; in fact, the assumption was often that the system was for data collection and/or that reports were ending up in a figurative black hole. However, physician M&M rounds may have used the IRS to populate the cases discussed, but the link between M&M rounds and the IRS was tenuous.

In contrast, OBS/NEO had many examples of learning that resulted directly from the IRS. For example, the accidental extubation project that the respiratory therapist spearheaded in OBS/NEO began with a recommendation to track the accidental extubations through the IRS. With a focus on reporting, and the commonality of the problem at other respected NICUs, the IRS became a more reliable way of knowing there was a problem, and that the intervention was successful. This hospital’s accidental extubation problem was known through analysis of incident reports, providing a clear linkage from the incident reports to a seemingly successful intervention.

### The influences

Litigation influenced incident reporting in both divisions, but in different ways. The GIM influence is the one traditionally reported in the literature: reluctance to report for fear of being sued or losing one’s licence [52, 53], although this is a bigger issue for physicians than for nurses [13]. However, as our data showed, litigation had the opposite effect in OBS/NEO. The threat of litigation is exceedingly high in these fields, and this serves as great motivation to avoid a lawsuit, and reporting practices are well established [19, 54]. However, this did not come about without some dedicated effort, specifically in training.

The introduction of the IRS in the two divisions, in terms of both customization and training on the IRS, may have been influenced by local ownership and the type of interaction with the front line practitioners. OBS had customized their form based on their previous experience – they knew what they wanted, and were able to approach the corporate project with their needs. Furthermore, they trained their users on “why” to report, instead of restricting the training to how to enter an event. This type of local ownership was termed “co-optation” by Waring & Currie [19], where the receiving group of a corporate project had the skills necessary to customize the corporate project to suit the needs of the local group and the larger organization. In GIM, with comparatively little local ownership of the system, the IRS was regarded more of a corporate project in which the customization of the IRS was invited by the corporate team, the training was corporately given and the training focussed principally on “how” to report. The importance of local ownership has been seen in the use of checklists; the World Health Organization endorses customizing the surgical safety checklist for local applicability [55], as has the Ontario Hospital Association [56]. The success of the Central Line Associated Blood Stream Infection (CLABSI) initiative was in part due to the customization of the checklist [47]. Local ownership was a key factor in the introduction of the IRS in OBS, but not in GIM.

Another key concept was the interaction with the healthcare professionals. If the corporate project allows the front line team to experiment and implement the intervention without being micro-managed, the front line team will be more interested in shepherding the intervention to be successful. As Reay et al. [57] found in their case study of institutional change, “When managers are able to encourage professionals to try new practices, and thus engage in quasi-independent meaning-making, the possibility of significant and sustainable changes in the nature of work becomes more feasible” (p987). This was also seen in the Michigan Keystone project, “The vertical core must focus on enabling teams to make changes, figuring out why some things are hard for staff, and making them easier to do” ([47], p. 195). While in this study, GIM was asked to participate in the introduction of the IRS, their contribution was driven by the corporate project. In contrast, OBS had a far more active role in driving how the IRS was introduced in their department, while still working within the wider framework of the corporate project. The type of involvement of GIM and OBS differed, which contributed to how the IRS was subsequently used in each division.

It is difficult to overestimate the influence of the MORE OB training, which has been shown to increase obstetrical outcomes and decrease insurance rates [58]. It had interdisciplinary teamwork as part of its fundamental philosophy. Interdisciplinarity has been identified as a major area of patient safety research where more improvement must be made [59, 60]. Weaver et al. [61] undertook a systematic review and found that interprofessional team training and/or communication initiatives are core elements of successful interventions to improve safety culture. Siassakos et al. [62] studied obstetrical interventions in team training, and found that early, direct and closed loop communication was critical for successful teams. Obstetrics is known for their leadership in team training [63]. Pronovost, Holzmueller, Ennen, & Fox [64] note that “The signs are hopeful that obstetrics and gynecology can make significant progress; this department is leading the field in team training” (p8). Obstetrics interventions also include concepts used in the aviation industry such as crew resource management [65] and simulation [66, 67]. Expanding the M&M meeting composition is another way to assure interdisciplinary collaboration. “If the team is provided a sufficiently diverse set of backgrounds, viewpoints, skills, and interests, then hidden assumptions are exposed; a broader repertoire of options, tactics, and tools is made available; tacit knowledge is made more explicit; and more interpretations and preferences are expressed” [68].

Team training has been seen as a critical ingredient in other patient safety initiatives. The very successful Michigan Keystone project that successfully tackled the problem of central line associated bloodstream infections included an interdisciplinary approach as one of its key factors.

Local improvement teams in the participating units were designed to not be dominated by any single profession but to have representatives from all stakeholder groups…Frequent interactions, reciprocal communication, and decentralization increase the social pressure to cooperate by reducing the social distance between members of unequal status and authority and strengthening the acceptance of group norms by the group members ([47], p. 182,183).

The importance of team training is a key factor for patient safety and incident reporting.

A specific dimension of team training is empowerment of those in the healthcare hierarchy who traditionally have not had a strong voice. MORE OB addressed this directly, ensuring that nurses, and later allied health professionals, were able to speak up if they felt something was going wrongly. This was another key component of the Michigan Keystone project, but this tool is very challenging to bring to the larger healthcare community. Reflecting on the participating hospitals in the initial Michigan Keystone project, Pronovost wrote

Perhaps most concerning is the response from nurses in participating hospitals when asked: “if a new nurse in your hospital saw a senior physician placing a catheter but not complying with the checklist, would the nurse speak up and would the physician comply?” The answer is almost always, “there is no way the nurse would speak up.” Doubly disturbing, physicians and nurses uniformly agree patients should receive the checklist items ([69], p. 204).

Empowering nurses is a critical aspect of patient safety, but it is far from trivial to sustain in routine healthcare practice.

## Conclusion

IRSs, while a key tool in patient safety, have been the subject of many studies, mainly focusing on reporting (or underreporting). This study of the whole process of incident reporting from detection to learning provides a more complex view. Furthermore, this study compared the processes at two hospital divisions, and revealed both the similarities and the differences between them. The model provided in Fig. 2 portrays these processes such that the two divisions can be directly compared, and also depicts the factors underlying the differences in the reporting processes.

Typical studies of IRSs focus on getting more events into the system [23–25]. However, more recent studies suggest that the number of reported events in an IRS is not a metric by which the system should be judged [29]. Pham et al. state that IRSs can be used to address local problems, and aggregate information for uncommon conditions. They recommend understanding and enhancing the analysis and system changing aspects to IRS, and providing meaningful feedback to the reporters who detect and submit reports. This study enhances understanding of these issues, by going beyond asking why people report or do not report and that interrogating the analysis and learning processes is integral to a successful IRS. This study also demonstrated a case of an uncommon condition (the accidental extubation of neonates) whose local existence was confirmed through the use of the IRS.

Many issues in safety are only analyzed once errors are found, or once something has failed [70–73]. This study was not prompted by a failure, nor was it an analysis of a system that was dysfunctional. The study of the normal, of the unremarkable, with an aim to explain the processes and understand the underlying dynamics is a new approach in safety [74, 75]. Studying when “things go right”, or safety II, is only now earning recognition in safety circles [76–78]. “Safety II is proactive, continuously trying to anticipate developments and events. It assumes that things, whether they go right or wrong, basically happen in the same way, regardless of the outcome” ([79], p. 239). This study helps promote this new focus in safety research by studying an IRS that seems to be functioning well.

The well-known Michigan Keystone initiative to reduce CLABSI (Central Line Insertion Bloodstream Infection) infections is a good case study of looking at a successful project to try to understand why it worked [47], and why a subsequent replication did not [45]. In these analyses, the authors note that the initiative is often trivially attributed to a short list of items, notably a checklist [46], which is an unfair depiction of the complexity of relationships and interactions that account for the programme’s success. Our study adds to this approach, by looking at the normal functioning of an IRS, with an aim to investigate factors that could account for how and why the system functions as it does in the two departments. In so doing, oversimplification of the factors was avoided, yet a concise explanation of why the IRS functions as it does was given.

This study has limitations. It took place in only one hospital, and was primarily restricted to interviews, albeit a wide variety of practitioners were interviewed to maximize the diversity of opinions on the IRS. This diversity exists in other healthcare environments (ICU, radiotherapy, surgery), so the views on the IRS and its functioning in the divisions are likely found in other healthcare domains. There may be other successful structures of an IRS that this study did not capture, given its limitation to two divisions. The findings from the divisions studied might not easily transfer to similar divisions in other hospitals, with different contextual factors. This study may not have captured all of the factors that influence the reporting processes in the two divisions examined, although those that were not described are likely to be less influential than the themes presented.

Practical implications of this study can be summarized in three broad categories. First, the importance of the interprofessional training seemed to be highly significant. Nembhard & Tucker [80] talked of healthcare traditionally using autonomous learning (experientially based) as opposed to deliberate learning (through specific training), and found that interdisciplinary collaboration promoted deliberate learning. This deliberate learning – interdisciplinary didactic sessions coupled with practice through simulations is quite costly, and serves as a non-trivial barrier to running courses like MORE OB. However, recently, medical insurers have paid for front line staff to participate in simulations, anticipating that the training will result in safer care, and thus fewer claims [81]. Medical insurers could play a more active role in encouraging these interprofessional learning opportunities.

A second area of practice implication is that of diversity. In his analysis of the values of highly successful healthcare organizations, Bohmer [82] highlights the importance of self-study and seeking dissenting views, the latter encouraged by Tucker & Edmondson [83] and Pronovost [69], especially encouraging nurses to speak up. The idea of having diverse opinions in groups responsible for patient safety is a key aspect to the success of the CLABSI Michigan Keystone project, in their conception of a Comprehensive Unit Safety Programme (CUSP) [84]. Diversity is also encouraged in M&M rounds [68]. The review of events by more than one profession allows a much richer understanding and well vetted recommendations for improvement [21, 32]. “Diversity of narratives can be seen as an enormous source of resilience in complex systems …The more angles, the more there can be to learn” ([85], p. 201). Encouraging more than one opinion and discussing with an aim to understand could help those involved in the IRS process bring a more holistic understanding to reported incidents.

A third area of practical implications deals with feedback. Leveson postulates that the main problem with safety in modern times is the lack of control or feedback loops allowing for those who initiate an action to learn its effects before an ultimate outcome [85]. Feedback has been identified as a critical IRS function [4], and the lack of feedback is seen as a barrier to reporting, as many IRSs fall short by not informing reporters of the effect of their efforts [23, 25, 51, 86]. This study adds to the literature in emphasizing the importance of feedback in IRSs, and suggests the importance of ensuring that feedback to the reporter is made an integral function of the IRS.

Finally, IRSs continue to be seen as important tools to improve patient safety. Yet, as has been shown, these systems can fall short of achieving the intended objectives. It is hoped that the findings from this study will be useful to academics and practitioners as they enrich their understanding of IRSs to enhance patient safety.

## Abbreviations

CAES, Canadian adverse events study; CLABSI, central line associated blood stream infection; CUSP, comprehensive unit safety programme; GIM, General Internal Medicine; ICU, intensive care unit; IRS, incident reporting system; M&M, mortality and morbidity; MORE OB, managing obstetrical risks effectively; NEO, neonatology; NICU, neonatal intensive care unit; OBS, obstetrics; OBS/NEO, obstetrics and neonatology; QA, quality Assurance; RCA, root cause analysis; UK, United Kingdom; WHO, World Health Organization
